# Lasing with Pumping Levels of Si Nanocrystals on Silicon Wafer

**DOI:** 10.1186/s11671-016-1707-z

**Published:** 2016-11-15

**Authors:** Wei-Qi Huang, Shi-Rong Liu, Zhong-Mei Huang, Xue-Ke Wu, Chao-Jian Qin, Qian-Dong Zhuang

**Affiliations:** 1 Institute of Nanophotonic Physics, Guizhou University, Guiyang, 550025 China; 2 State Key Laboratory of Surface Physics, Key Laboratory of Micro and Nano Photonic Structures (Ministry of Education) and Department of Physics, Fudan University, Shanghai, 200433 China; 3 State Key Laboratory of Ore Deposit Geochemistry Institute of Geochemistry, Chinese Academy of Science Institute of Geochemistry, Guiyang, 550003 China; 4 Physics Department, Lancaster University, Lancaster, LA1 4YB UK

**Keywords:** Silicon nanocrystal, Pulsed laser deposition, Visible lasing, Four-level system

## Abstract

It is reported that the silicon nanocrystals (NCs) are fabricated by using self-assembly growth method with the annealing and the electron beam irradiation processes in the pulsed laser depositing, on which the visible lasing with higher gain (over 130 cm^−1^) and the enhanced emission in optical telecommunication window are measured in photoluminescence (PL). It is interesting that the enhanced visible electroluminescence (EL) on silicon nanocrystals (Si-NCs) is obviously observed by the naked eyes, and the light-emitting diode (LED) of the Si-NCs with external quantum efficiency of 20% is made on silicon chip in our laboratory. A four-level system is built for emission model in nanosilicon, in which the PL and EL measurement and transmission electron microscope (TEM) analysis demonstrate that the pumping levels with shorter lifetime from the rising energy of the Si quantum dots due to the quantum confinement effect occur, and the electronic localized states with longer lifetime owing to impurities bonding on Si-NCs surface are formed in the crystallized process to produce the inversion of population for lasing, where the optical gain is generated.

## Background

The prominent position of silicon follows from a unique combination of advantageous properties such as the availability of larger single crystals, higher purity, effective conductivity engineering, a matching insulator, and importantly natural abundance. Even though the optical properties of crystalline silicon are relatively poor due to its indirect bandgap precluding the efficient emission, silicon light source is of key importance for integrated Si optoelectronics material and devices [1–6], in which as a potential material for Si light source, silicon nanocrystals (Si-NCs) have received a lot of interest concerning enhancements of their emission [7–16].

It has been established that the spectrum of the photoluminescence (PL) emission from Si-NCs shifts to the blue due to the quantum confinement (QC) effect related to the rising electronic states which have shorter lifetime (in nanosecond (ns) scale) [17, 18] to be suitable for pumping levels, due to the Heisenberg principle related to ⊿*t* ~ *h*/⊿*E* [19] in the QC effect. The results of experiment and calculation indicate the quantization-related open of the bandgap and the enhancement of the radiative recombination rate, as momentum conservation is gradually relaxed with decrease of nanocluster size.

On the other hand, the impurities of oxygen and nitrogen or the interface and defects lead to the forming of the electronic localization states into the bandgap due to broken symmetry of system, whose energy position refers to Si–O and Si–N bonds on curved surface (CS) related to smaller nanoclusters called CS effect [19, 20]. Here, the electronic localization states have a longer lifetime (in microsecond (μs) scale) [21, 22] to be suitable for producing inversion of population in the levels. Therefore, in the article, the energy band becomes discrete for the “band” conception to cease instead of building a four-level system involving the QC electronic states such as the pumping level from the Si-NCs, the higher emission level of the electronic localized states due to n-doped on the Si-NC surface, the lower emission level of p-doped states, and the valence band level.

We fabricate the silicon nanocrystals by using the pulsed laser deposition (PLD) method with the annealing and the electron beam irradiation processes [23] in which the annealing time and the plasmonic interaction play important roles [24]. The photoluminescence (PL) and the electroluminescence (EL) measurement and the transmission electron microscope (TEM) analysis in the Si-NC structures demonstrate that the pumping states with a shorter lifetime from the Si quantum dots (QDs) occur, which are verified in their direct emission in shorter wavelength with the QC effect, and on the other hand, the electronic localized states with longer lifetime owing to impurities bonding on the Si-NCs surface are formed in the crystallized process to produce the inversion of population for lasing, in which the optical gain in PL emission is measured and the visible emission in EL on Si-NCs is obviously observed by the naked eyes. The light-emitting diode (LED) of Si-NCs with external quantum efficiency of 20% is made on silicon chip in our laboratory.

The methods for fabricating the Si-NCs are self-assembly from silicon-rich silicon oxide matrices [25–28], plasma synthesis [29, 30], and colloidal chemistry [31, 32] in tradition. The successful self-assembly growth method with the annealing and the electron beam irradiation processes on the amorphous films prepared by PLD process is better to form the silicon nanocrystals in narrower size distribution and is easy to control the impurities on the Si-NC surface, which are in the pure-physics processes with less factors disturbing the fabrication.

## Experiments and Results

**Fig. 1:**
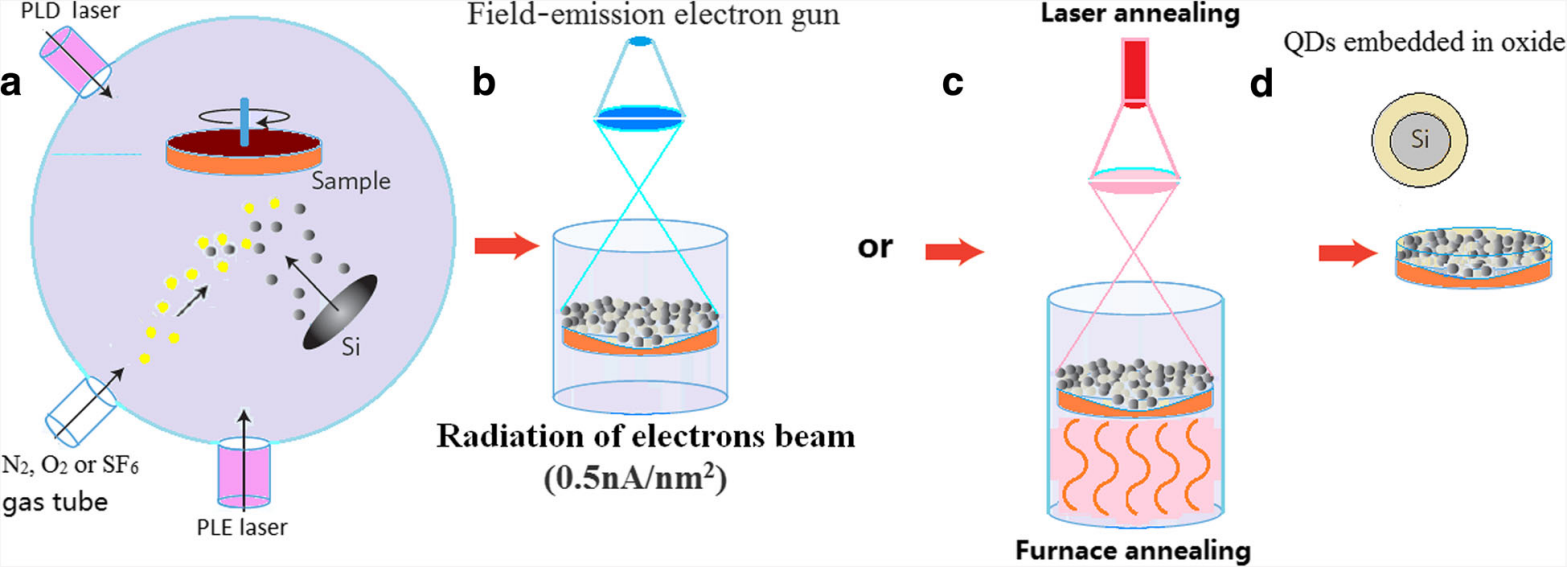
**a** Combination fabrication system with pulsed laser etching (PLE) and pulsed laser deposition (PLD) devices. **b** Irradiation system of electron beam, in which the silicon film is exposed under electron beam with 0.5 nA/nm^2^ in Tecnai G2 F20 system for crystallization. **c** Furnace annealing device and laser annealing device. **d** Formation of Si QDs with the structure of the phase separation between Si and SiO_*x*_ (with *x* < 2) or Si_*y*_N_*x*_ (with *x* < 4 and *y* > 3)

A silicon wafer of P-type (100) oriented substrate of 1–10 Ωcm is taken on the sample stage in the combination fabrication system with pulsed laser etching (PLE) and PLD devices, as shown in Fig. 1a. A pulsed Nd:YAG laser (wavelength 1064 nm, pulse length 60 ns FWHM, repetition rate 1000) is used to etch the Purcell micro-cavity on Si sample in the PLE process. In the cavity, a third harmonic of pulsed Nd:YAG laser at 355 nm is used to deposit amorphous silicon film in the PLD process. Then, the amorphous silicon film is exposed under electron beam with 0.5 nA/nm^2^ for 10~30 min in Tecnai G2 F20 system for crystallization (Fig. 1b), in which the electron beam from the field-emission electron gun, accelerated by 200 kV, are in higher energy and in better coherent status, whose electronic de Broglie wave in nanoscale is suitable for producing resonance to transfer energy to atoms for crystallizing. The crystallizing time under the electron beam irradiation is about 15 min to form the Si QDs embedded in the amorphous silicon. The impurity process through gas tube is involved in PLD, such as nitrogen or oxygen gas. After radiation under the electron beam for 15 min [23], the Si QD structures are built and embedded in the SiO_*x*_ (with *x* < 2) or the Si_*y*_N_*x*_ (with *x* < 4 and *y* > 3) amorphous film. The method of electron beam irradiation is a new and interesting way to fabricate the Si-NC structures.

Another interesting method for fabricating the silicon nanocrystals is in the photons interaction. In Fig. 1c, at first, the amorphous silicon film is formed in the PLD process. Subsequently, a pulsed Nd:YAG laser is used to irradiate the amorphous silicon film, in which the crystallizing process for producing QDs forms by controlling interaction of the photons and the plasmonic vibration called laser annealing. In the physical process, the standing wave and the lattice pattern with characteristic of the Wigner crystal [33, 34] in the plasmonic vibration are observed. In the furnace annealing, high-temperature annealing of the substoichimetric film (typically 900~1100 °C) produces a phase separation between Si and SiO_*x*_ (with *x* < 2) for forming Si QDs (Fig. 1d). The dimensions, crystallinity, and size distribution of the Si-NCs depend on the Si excess, the temperature, and the annealing time.

**Fig. 2:**
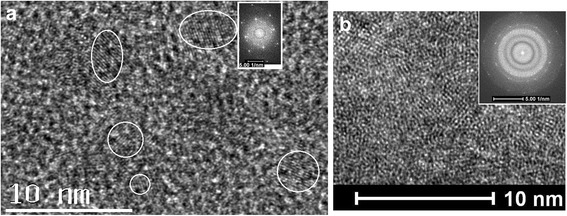
**a** TEM image of Si QDs embedded in amorphous silicon, in which the inset shows the FFT image of crystal in QDs related to crystallizing time of 15 min under electron beam irradiation. **b** Random bigger bulks of crystals produced after electron beam irradiation for longer time (30 min), in which the inset shows the FFT image of crystal in the bulks

In Fig. 2a, the TEM image shows QD structure of the Si-NCs embedded in the amorphous silicon, whose size distribution range is from 1.2 to 5 nm related to the crystallizing time of 15 min under the electron beam irradiation, in which the right inset shows the electron diffraction pattern of crystal in the QDs. It is very interesting that the crystal structures become bigger and bigger with the increase of crystallizing time to break the QD structures and form some random bigger bulks of crystals, and the crystal structures prepared under the electron beam irradiation for 30 min are shown in the TEM image of Fig. 2b, in which the inset shows the FFT image related to the electron diffraction pattern in the crystal structures. These crystallizing processes can be observed in the TEM analysis.

**Fig. 3:**
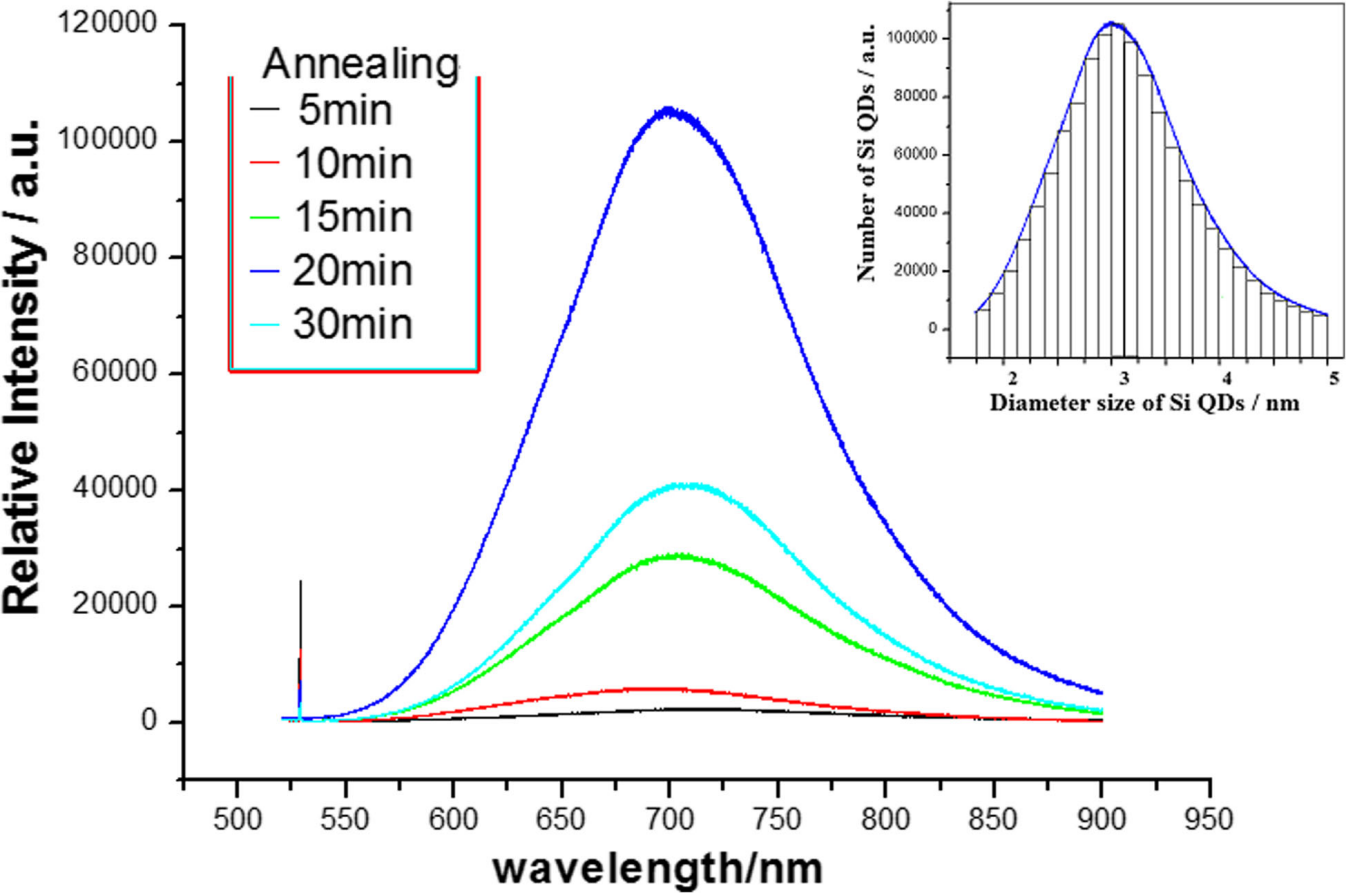
PL spectra on Si-NC samples, in which the intensity evolution of wavelength center near 700 nm occurs with change of furnace annealing time, and the optimum annealing time may be about 20 min for producing more numbers of QDs in various scales whose size distribution is shown in the inset

In the same way, the annealing time plays an important role to control crystallizing for forming QD structures in laser annealing or furnace annealing. The Si QD emission in the QC effect is observed in Fig. 3, in which the intensity evolution of wavelength center near 700 nm occurs in the PL spectra on the Si-NC samples with change in furnace annealing time. Here, the optimum annealing time may be about 20 min for producing more numbers of QDs in various scales whose size distribution is shown in the inset of Fig. 3. This optimum annealing time is related to the suitable crystallizing process for forming Si QDs to increase PL emission.

**Fig. 4:**
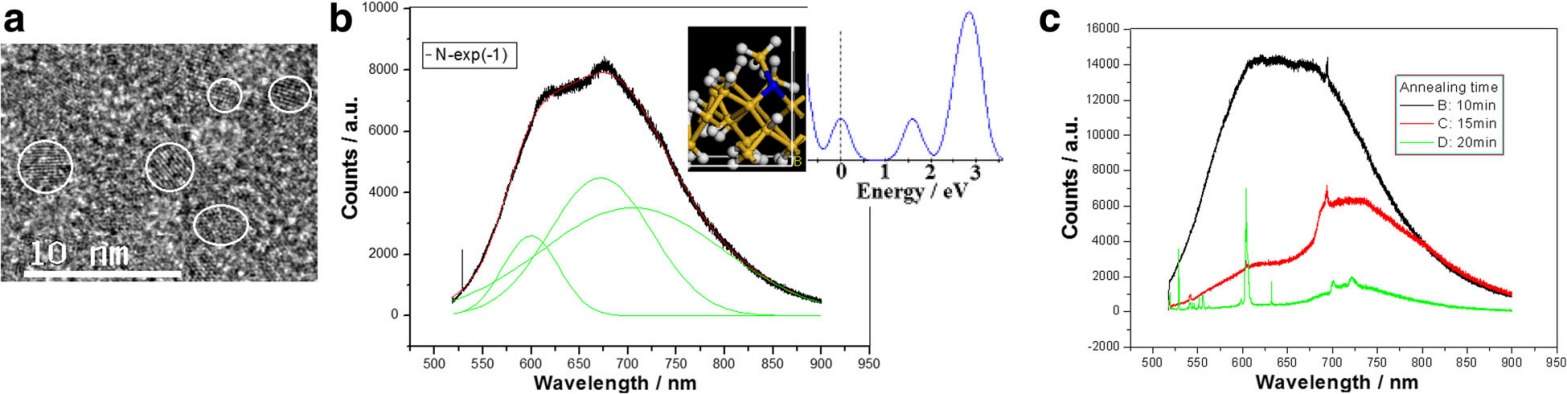
**a** TEM image of the Si QD structures prepared in nitrogen environment of 0.1 Pa. **b** PL spectrum on the Si-NCs doped with nitrogen, whose bonds structures and density of states in simulation are shown in the inset. **c** PL spectra measured on the Si QD structures doped with nitrogen after furnace annealing in different annealing time

The TEM image in Fig. 4a shows the Si QD structures prepared in nitrogen environment of 0.1 Pa. The nitrogen impurities and the Si-N bonding on the surface of Si QDs lead to the forming of the electronic localization states into bandgap due to broken symmetry of system, which is related to the fit peaks near 580 and 670 nm, and the fit peak band from 600 to 850 nm refers to direct emission from QDs not used for pumping in the PL spectrum, as shown in Fig. 4b. Their bond structures and the density of states in simulation are shown in the inset of Fig. 4b. In Fig. 4c, we make a comparison with the PL spectra measured on the Si QD structures doped with nitrogen after furnace annealing in different annealing times, which indicates that the suitable annealing time can generate the sharper peak from the localization states due to Si-N bonds on Si QDs.

**Fig. 5:**
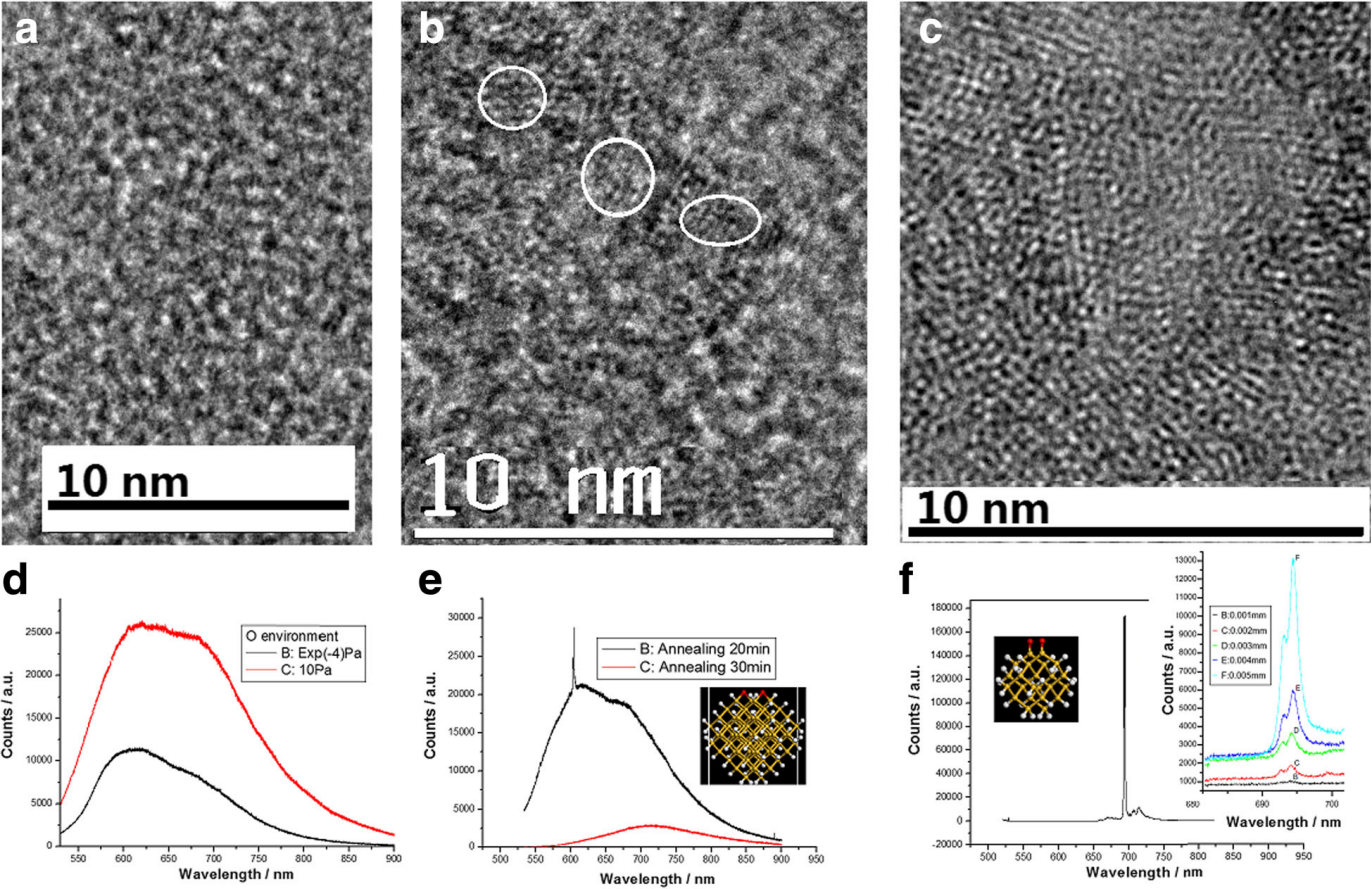
Fabrication process of the Si QD structures doped with oxygen and their emission. **a** TEM image of the amorphous structures on Si film prepared by using PLD method in oxygen environment. **b** TEM image of the Si QD bonding with oxygen atoms on surface after irradiation of electron beam for 15 min. **c** TEM image of the Si crystals doped with oxygen after irradiation of electron beam for 30 min, in which the crystallization process is almost finished. **d** PL spectra measured on the Si-NC structures doped with oxygen after furnace annealing for 5 min, in which it is noted that the emission intensity near 600 nm is enhanced on the sample prepared in dilute oxygen of 0.0001 Pa, and the intensity increases near 700 nm on the sample prepared in concentrated oxygen of 10 Pa. **e** PL spectra measured on the Si-NC structures prepared in dilute oxygen for forming Si–O–Si bond as shown in the inset, in which the sharper peak near 600 nm is observed after annealing for 20 min, but it disappears after annealing for 30 min. **f** PL spectra measured on the Si-NC structures prepared in concentrated oxygen for forming Si=O bond after annealing for 20 min, in which the very sharper peak near 700 nm with higher gain (over 130 cm^−1^) and full width at half maximum of 0.5 nm is measured as shown in the inset

The TEM image of Fig. 5a shows the amorphous film structures prepared by using PLD method in oxygen environment, in which the Si QDs bonding with oxygen atoms on the surface can be obtained after irradiation of electron beam for 15 min (in the TEM image of Fig. 5b), and the crystallization is almost finished after irradiation of electron beam for 30 min (in the TEM image of Fig. 5c). The PL spectra are measured on the Si-NC structures doped with oxygen after furnace annealing for 5 min as shown in Fig. 5d, in which it is noted that the emission intensity near 600 nm is enhanced on the sample prepared in the dilute oxygen of 10^−4^ Pa, and the intensity increases near 700 nm on the sample prepared in the concentrated oxygen of 10 Pa.

These phenomena may be related to the electronic localized states at 2 eV from Si–O–Si bridge bonds formed in dilute oxygen and the electronic localized states at 1.78 eV from Si=O bonds formed in concentrated oxygen [19]. After increasing annealing time to 20 min, the sharper peak near 600 nm in the PL spectrum is observed on the Si-NC structures prepared in dilute oxygen in which Si–O–Si bonds are easy to generate on the Si QDs, but they gradually disappear after annealing for 30 min, as shown in Fig. 5e. It is interesting that the very sharper peak near 700 nm with the higher gain (over 130 cm^−1^) and the full width at half maximum of 0.5 nm is measured on the Si-NC structures in the Purcell cavity prepared in concentrated oxygen for forming Si=O bond after annealing for 20 min, as shown in Fig. 5f, and the inset shows the evolution of the peak intensity as a function of the optically pumped sample length measured by using the various stripe length method for getting optical gain. Here, the Purcell cavity prepared by the PLE process is important for resonating and selecting mode of emission in Fig. 5f.

**Fig. 6:**
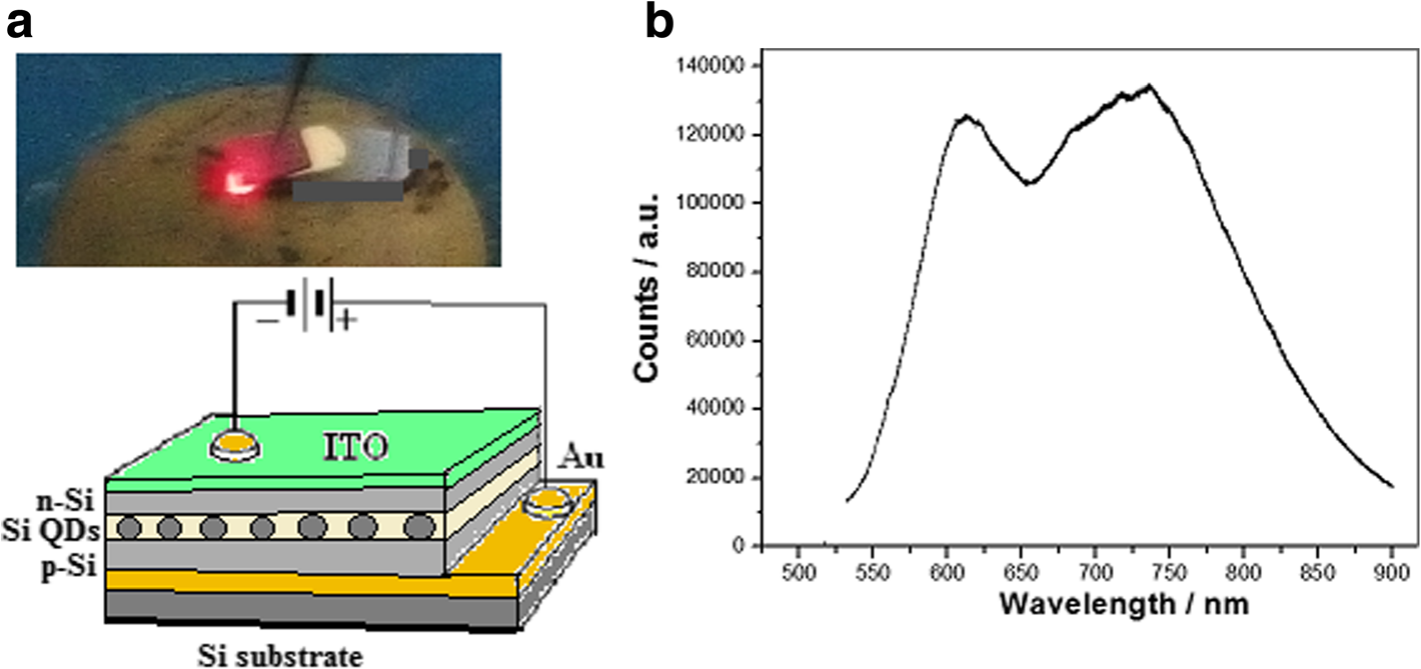
A LED device with the Si QDs doped oxygen built on silicon wafer. **a** Construction of the LED device and its emission image. **b** Spectrum on the LED emission

A LED device with the Si QDs doped with oxygen is built on silicon wafer, whose external quantum efficiency in EL emission is over 20% and the threshold current is about 50 mA/mm^2^. The device construction is shown in Fig. 6a, in which the bright light is observed on the LED by the naked eyes and its emission spectrum is shown in Fig. 6b.

## Results and Discussion

**Fig. 7:**
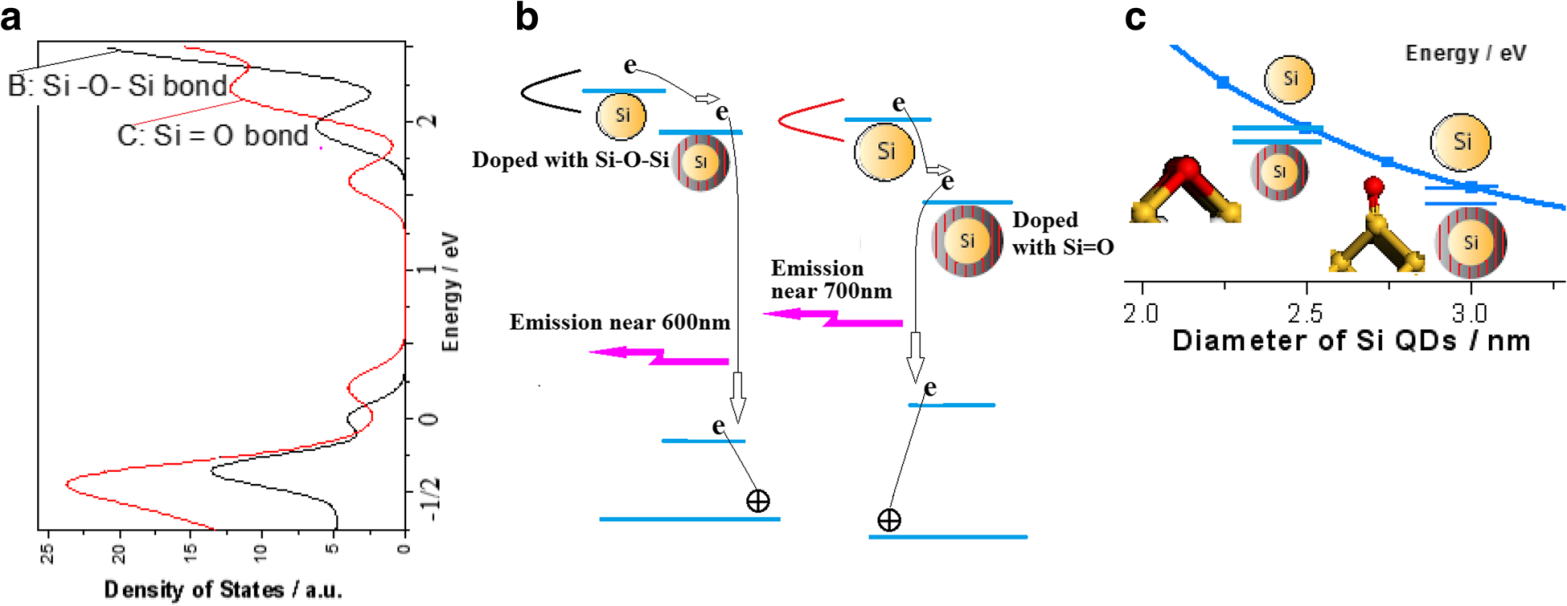
Emission model construction with the four-level system. **a** Density of states from simulation calculation related to the electronic localized states owing to Si–O–Si or Si=O bonding on Si-NC surface. **b** Construction of the four-level system with the pumping levels on the Si QDs and the trapping states of the QDs doped with oxygen. **c** Energy position related to the pumping levels on the Si QDs by the QC curve and the trapping states of the QDs doped with oxygen by the CS effect

An emission model with a four-level system involving the QC electronic states from the Si-NCs and the electronic localized states due to the n-doped Si-NCs on the surface is built, as shown in Fig. 7. We have chosen some model structure in order to simulate the experiment process. The electronic behavior is investigated in the work by an ab initio non-relativistic quantum mechanical analysis. The DFT calculation were carried out by using the local density approximation (LDA) and non-local gradient-corrected exchange-correlation functional (GGA) for the self-consistent total energy calculation. In Fig. 7a, the density of states from simulation result provides the construction of the four-level system, in which it is noted that the electronic localized states due to the Si–O bonding on the Si-NC surface are under the Si QD states as shown in Fig. 7b. It is interesting that the position of the electronic localized states from Si=O bond is deeper than that from Si–O–Si bond in the gap. Figure 7c shows the QC effect with change of QD size and the trapping states of the QDs doped with oxygen.

**Fig. 8:**
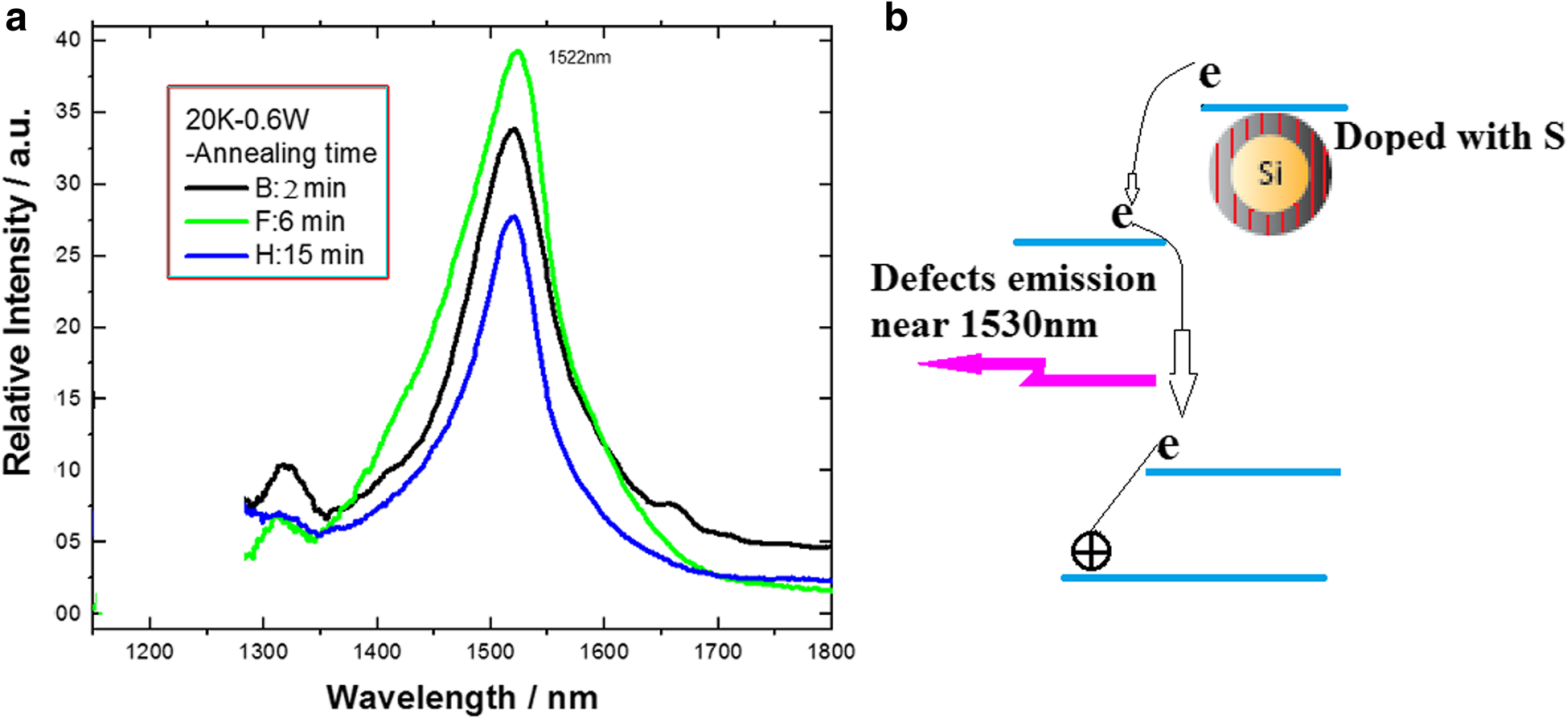
**a** PL spectra with the annealing effect measured on the Si-NC structures with S impurity and **b** its emission model in optical telecommunication

In the same way, the enhanced peak near 1530 nm in the PL spectra is measured on the Si-NC structures with sulphuric impurity at 20 K, in which it is interested that the annealing time plays an important role for producing the enhanced defect emission, as shown in Fig. 8a. And the furnace annealing time of 6 min is better to form defect structures for emission, which is related to the initial period for more defects in crystallization process. Figure 8b shows an emission model in an optical telecommunication window with the four-level system, in which the levels from the defect states with longer lifetime than those in the Si-NC states can form the inversion of population for enhanced emission.

## Conclusions

We fabricate the silicon nanocrystals by using a self-assembly growth method with the annealing and the electron beam irradiation processes on the amorphous silicon film prepared by using PLD. The silicon nanocrystals have been one of the most important developments in the field, as they offer strong carrier confinement and modification of the energy levels through quantization, as well as the ability to use their surface as a further design parameter, from which we provide a emission model with the four-level system. In the PL, EL measurement and the TEM analysis on the Si-NC structures, it is demonstrated that the pumping levels with higher energy due to the QC effect of Si QDs and the electronic localized states with longer lifetime owing to impurities bonding on Si-NC surface are produced in the crystallized process for the inversion of population in lasing. The optical gain in PL emission is measured in the Si-NC structures, and the visible EL on the Si-NCs is obviously observed by the naked eye. The light-emitting device on the Si-NCs with external quantum efficiency of 20% is made on silicon chip in our laboratory. If one could have better controlling for the nature of the photoactive impurities on the Si-NCs or the photoactive defects in Si crystallization, and increase their density for building the inverse population levels, thereby increasing the spectral density of emission, a true all-silicon laser will be in reach on Si chip.

## Methods

### Fabrication of Silicon Quantum Dots Under Irradiation of Electron Beam

The amorphous silicon film was exposed under electron beam with 0.5 nA/nm^2^ for 5~30 min in Tecnai G2 F20 system, in which coherent electron beam from field-emission electron gun, accelerated by 200 kV, has higher energy and better coherence. After irradiation under electron beam for 15 min, the silicon quantum dot (Si QD) structures are built and embedded in SiO_*x*_ (with *x* < 2) or Si_*y*_N_*x*_ (with *x* < 4 and *y* > 3) amorphous film prepared in oxygen or nitrogen gas, respectively, in the PLD device. Physical process of fabricating Si QDs under irradiation of electron beam is shown in Fig. 1, in which it is interesting that the Si QDs with the spherical shape gradually grow up after irradiation of electron beam for suitable time, whose size range is from 2 to 5 nm.

### Laser Annealing Process

A pulsed Nd:YAG laser at 1064 nm is used to make annealing on the amorphous film of silicon, in which the temperature sensor indicates that the temperature is about 900 °C on the area exposed under laser beam. In the laser annealing process, besides the thermal action, it is important that coupling between photons and plasmons produced by nanosecond pulsed laser on Si surface forms resonance to transfer the energy to the atoms for crystallizing. It is very interesting that the lattice pattern of plasmonic standing wave is observed in coupling between photons and plasmons as shown in the inset of Fig. 1c, which is similar with the Wigner crystal structure.

### Electroluminescence Measurement on the LED with Si QDs

The construction of silicon QD LED is shown in Fig. 6a, in which the PIN structure involves the bottom buffer silicon layer, the top Si layer doped with sulfur, and the medium layer with Si QDs doped with oxygen. In the LED device, the positive pole is connected on the gold film under the P-type Si layer and the negative pole is connected with the ITO film on the N-type Si top layer. The electroluminescence (EL) spectra is measured on the LED, in which the bright light is observed by the naked eyes, whose external quantum efficiency in PL emission is over 20% and the threshold current is about 50 mA/mm^2^.
